# Long-Term Effects of Social Insurance on Adult Mortality: Evidence From Three Social Programs in Mexico

**DOI:** 10.1093/geroni/igab046.1923

**Published:** 2021-12-17

**Authors:** Emma Aguila, William Dow, Susan Parker

**Affiliations:** 1 Sol Price School of Public Policy, University of Southern California, California, United States; 2 UC Berkeley, Berkeley, California, United States; 3 University of Maryland, College Park, Maryland, United States

## Abstract

Research on the mortality effects of social insurance programs for older adults has generated conflicting results. Some studies suggest important health benefits, others find no effects, and still others find unintended adverse effects potentially linked to pathways such as increased obesity. Evidence has focused predominantly on short-run effects rather than net long-run mortality effects and their effects on the health of older adults has been particularly understudied. Mexico offers a unique opportunity for studying the long-run effects of social programs on adult mortality. Within a ten-year period, Mexico introduced the following influential social insurance programs: Progresa conditional cash transfer (CCT) program in 1997, 70 y más unconditional cash transfer (UCT) program for older persons in 2007, and Seguro Popular, a public health insurance program (PHI) for the uninsured, in 2004. In this paper we analyze effects on mortality for middle-age and older adults, by gender, 10-20 years after program implementation.

